# Plant Cell Wall Changes in Common Wheat Roots as a Result of Their Interaction with Beneficial Fungi of *Trichoderma*

**DOI:** 10.3390/cells9102319

**Published:** 2020-10-19

**Authors:** Aneta Basińska-Barczak, Lidia Błaszczyk, Kinga Szentner

**Affiliations:** 1 Department of Pathogen Genetics and Plant Resistance, Institute of Plant Genetics, Polish Academy of Sciences, 60-625 Poznan, Poland; lgol@igr.poznan.pl; 2 Department of Chemistry, Poznan University of Life Sciences, Wojska Polskiego 75, 60-625 Poznan, Poland; kinga.szentner@up.poznan.pl

**Keywords:** wheat, *Trichoderma*, plant cell wall, immunodetection, confocal microscopy, scanning electron microscopy

## Abstract

Plant cell walls play an important role in shaping the defense strategies of plants. This research demonstrates the influence of two differentiators: the lifestyle and properties of the *Trichoderma* species on cell wall changes in common wheat seedlings. The methodologies used in this investigation include microscopy observations and immunodetection. In this study was shown that the plant cell wall was altered due to its interaction with *Trichoderma*. The accumulation of lignins and reorganization of pectin were observed. The immunocytochemistry indicated that low methyl-esterified pectins appeared in intercellular spaces. Moreover, it was found that the arabinogalactan protein epitope JIM14 can play a role in the interaction of wheat roots with both the tested *Trichoderma* strains. Nevertheless, we postulate that modifications, such as the appearance of lignins, rearrangement of low methyl-esterified pectins, and arabinogalactan proteins due to the interaction with *Trichoderma* show that tested strains can be potentially used in wheat seedlings protection to pathogens.

## 1. Introduction

Common wheat (*Triticum aestivum* L.) is one of the most widely cultivated crops in the world. Statistics indicate that in 2018–2019, global wheat production was 762.4 million tons [1]. However, wheat production is mainly affected by the incidence of foliar and roots disease caused by pathogens, such as fungi from different genera, including *Fusarium*, *Puccinia*, *Bipolaris sorokiniana*, *Zymoseptoria tritici*, and others [2]. From an environmental perspective, it would be beneficial to use biological control agents (BCAs), serving as natural antagonists to phytopathogens [3]. The most studied and promising microorganisms used in a biocontrol system are the *Trichoderma* species [4,5]. *Trichoderma* strains used as biocontrol agents created a positive impact on plants, nutrient uptake, fertilizer use efficiency, growth, and rate of seed germination. These fungi triggered induced systemic resistance (ISR) in monocots [6,7] and dicots [8,9,10]. Data showed that these fungi can also stimulate a systemic acquired resistance (SAR) in plants [11]. Fungi of the genus *Trichoderma* (teleomorph *Hypocrea*) colonize different ecological niches and are free-living organisms. Habitats occupied by these fungi include rotting wood, soil, and the rhizosphere [4,12,13]. *Trichoderma* spp. influence pathogens, such as oomycetes (*Oomycota*), bacteria, viruses, and pathogenic fungi via hyperparasitism, competition, and antibiosis [14,15]. Our recent studies have revealed that *Trichoderma* spp. can suppress mycotoxin production by *Fusarium* species on natural substrates, exhibit mycoparasitic behavior, such as coiling on pathogen hyphae, and prohibit pathogen growth [16]. Moreover, *Trichoderma* spp. can also produce numerous secondary metabolites and lytic enzymes. Owing to these abilities, these fungi can interact with plants and other microorganisms or exist as a saprotroph on decaying wood. These fungi can also produce volatile metabolites, including 6PAP (6-n-pentyl-6H-pyran-2-on, 6PP). This compound impacts plant growth and leads to the development of systemic resistance [17,18], while displaying antifungal properties [19,20,21,22]. In our previous study, we reported that the production of volatile metabolites is species- and strain- dependent, and we identified the *Trichoderma atroviride* strain AN35 as the most efficient producer of 6PAP and other volatile metabolites [23]. Moreover, we demonstrated that the *Trichoderma cremeum* strain AN392, isolated from decaying wood, was the best producer of cellulases (EC3.2.1) and xylanases [24].

It is well known that plant cell walls play multiple roles. The cell wall is a structure comprising polysaccharide polymers like pectin, hemicellulose, cellulose, and nonpolysaccharide polymers like lignin. Enzymatic and structural proteins are also a part of plant cell walls [25,26]. Like plants, the plant cell walls are also influenced by environmental conditions and other organisms. This is a dynamic structure that changes under different conditions. It has been demonstrated that specific molecules of plant cell walls play a role in shaping the defense strategies of plants. The sensitivity of plants to pathogens can be determined by the level of methyl esterification of pectins. A high level of methyl-esterified homogalacturonan (HG) is equivalent to an increase in plant resistance [27,28]. Oligogalacturonides, endogenous molecules, or fragments of homogalacturonan play a role in enhancing plant response to pathogens [29,30]. Similarly, xylans can have an impact on the resistance and sustainability of plants to fungal pathogens. Studies have shown that a higher amount of xylans in the cell walls of *Arabidopsis* and barley mutants increased the resistance of those plants to fungal pathogens [31]. Studies have shown that lignins are involved in a plant’s defense strategy, making the cell wall more resistant to cell-wall-degrading enzymes (CWDEs) and diffusion of toxins produced by pathogens [32,33,34]. Proteins, such as arabinogalactan proteins (AGPs), constitute an important component of plant cell walls. They can be involved in many plant processes, such as the regular growth and development of plant organs, including roots [35] and the plasticity of the cell wall [36]. They also initiate the interaction between a plant and symbiotic or pathogenic microorganisms. It has been shown that they regulate the interaction with symbiotic microorganisms such as bacteria and mycorrhiza fungi. Arabinogalactan proteins are involved in all of the stages of the interaction between roots and microorganisms, as well as in the selection of microorganisms and the immobilization of pathogenic microorganisms [37]. Arabinogalactan proteins are widespread in the plant kingdom and are found in both bryophytes and angiosperms [38]. Many studies have been conducted on the changes in plant cell walls during their interaction with microorganisms. However, there is limited knowledge about the participation of plant cell wall components in the interaction of common wheat plants with *Trichoderma* fungi. Hence, this study aimed to assess the changes occurring in the cell wall of common wheat (*T. aestivum* L.) seedlings after their interaction with *T. cremeum* and *T. atroviride*, differentiated with their properties, such as production of secondary metabolites and lifestyles. The analysis of plant cell wall changes was performed by microscopy observations: scanning electron microscopy, light microscopy, and confocal microscopy with immunocytochemistry, where monoclonal antibodies were used.

## 2. Materials and Methods

### 2.1. Plant Material

The study material included wheat seedlings (*T. aestivum* L.) of the cultivars of spring wheat (Bombona developed by Danko Plant Breeding Ltd. Co. (Choryn, Poland)) and winter wheat (Legenda developed by Poznań Plant Breeders Ltd. (Tulce, Poland)).

#### 2.1.1. Plant Growth Conditions

Wheat seeds were sterilized using 0.5% active chlorine for 5 min and then washed 3 times by autoclaved deionized water. Seedlings were germinated for 3–4 days on solidified agar. After this duration, the wheat seedlings were transferred to semifluid agar vials of 30 mL capacity, which were partially inoculated with suitable species of *Trichoderma*.

#### 2.1.2. Fungal Material

The fungal material used for the research were the *T. atroviride* strain AN35, and the *T. cremeum* strain AN392, isolated and previously characterized by Błaszczyk et al. [16,24,39], Jeleń et al. [23], and Marecik et al. [40]. These species are deposited in the collection of the Institute of Plant Genetics, Polish Academy of Science (curators of the collection: Lidia Błaszczyk, Jerzy Chełkowski), and in The Westerdijk Fungal Biodiversity Institute (CBS-KNAW), Utrecht, Netherlands (for *T. atroviride* AN35).

#### 2.1.3. Inoculum Preparation and Plant Inoculation

Pure cultures of *T. atroviride* AN35 and *T. cremeum* AN392 were recultured from stock slants to 9 cm diameter Petri dishes with potato dextrose agar (PDA medium, Oxoid™ Thermo Fisher Scientific, Waltham, MA, USA). The fungal material was incubated at 23 °C in 12 h/12 h darkness/light conditions. The inoculum constituted only spores (without PDA disc) from an area of 5 mm of diameter from the 7-day old culture. Each plant treated with *Trichoderma* was inoculated with spores from 1 area. Control and inoculated with fungi plants were grown for 14 days at a temperature of 23 °C under 12 h/12 h darkness/light conditions in a growth chamber. After this time, the material was sampled. All of these steps were carried out under sterile conditions.

### 2.2. Microscopy Observations

**Figure 1:**
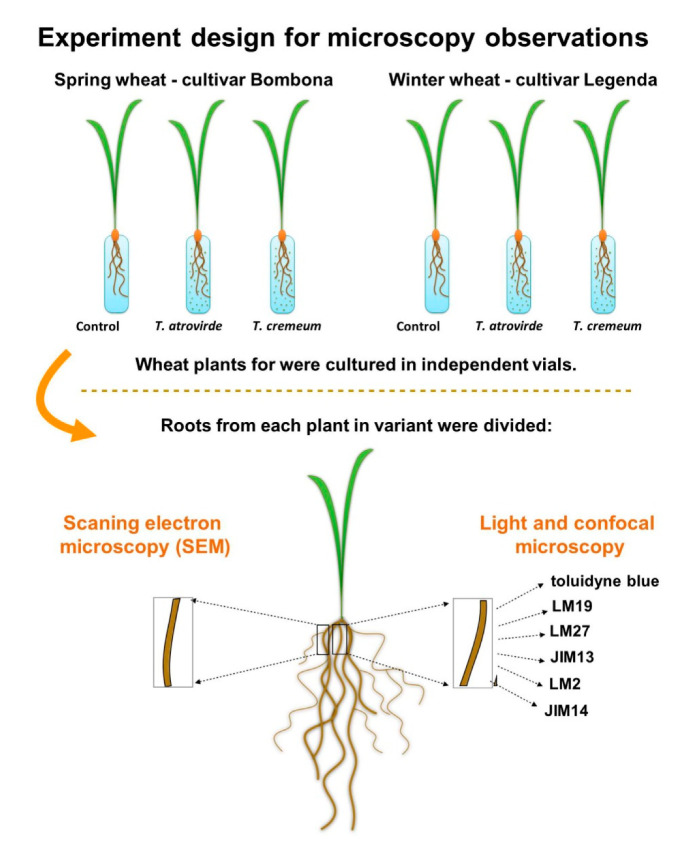
Scheme of experiment for microscopy observations.

Each variant for microscopy observations were replicated from 7 to 10 plants. Roots from this same plant root system were divided—one (root hairs zone) for light and confocal microscopy. Three sections were taken for each staining. Second root (root hairs zone) were taken for scanning electron microscopy (SEM). Figure 1 shows a scheme of the experiment.

#### 2.2.1. Preparation of Plant Material for Electron Scanning Microscopy

After 14 days of incubation with *Trichoderma* spp., the roots of wheat seedlings were fixed in a mixture of 4% methanol-free formaldehyde (Polysciences, Hirschberg an der Bergstrasse, Germany), 0.5% glutaraldehyde (Polysciences, Hirschberg an der Bergstrasse, Germany), and phosphate-buffered saline (PBS) at 4 °C for 24 h. SEM measurements were realized using the Quanta FEG 250 (FEI) microscope under low vacuum mode at the pressure of 70 Pa. Low vacuum conditions were realized by the dosing of ultra-pure water vapor. This prevents the samples from charging, thus, no coating was used. The plant material was frozen in liquid nitrogen and lyophilized for 4 h. SEM imaging was realized using 10 kV beam acceleration voltage with the 30 µm aperture, at a typical working distance of 10 mm.

#### 2.2.2. Preparation of Plant Material for Light and Confocal Microscopy Observations

After 14 days of inoculations, the roots of the wheat seedlings were fixed as mentioned above. Then, the material was first dehydrated in an ethanol series (10%, 30%, 50%, 70%, 80%, 90%, 96%, 100%), then dehydrated in a solution containing 100% acetone and embedded in Technovit 8100 resin (Kulzer GmbH, Wehrheim, Germany) in line with the manufacturer’s instructions. The samples were sectioned on a rotary microtome (Leica, RM2125RTS) on 5 µm thin sections. Transverse root sections were collected on microscope slides coated with poly-l-lysine (Merck KGaA, Darmstadt, Germany, formerly Sigma, St. Louis, MO, USA).

#### 2.2.3. Detection of Lignins, Pectins, and Colonization of Wheat Roots with Trichoderma Hyphae

The staining procedure of wheat roots treated with *Trichoderma* was carried out according to Chacón et al. [41]. The sections of roots were treated with 0.5% toluidine blue solution (Kolchem, Lodz, Poland) dye for 3 min, for detecting lignin, pectin, and hyphae of *Trichoderma* inside the wheat roots. The observations were captured using a light microscope (Olympus CX-41-1 with UC-30 camera, Olympus, Japan). Pectins were dyed a red–purple color, lignins a blue color.

#### 2.2.4. Immunolabeling of Pectins, Hemicellulose, and Arabinogalactan Proteins

The detection process was carried out in sections using primary monoclonal antibodies—LM19 epitope (PlantProbes, Leeds, UK, Table 1) for low methyl-esterified pectins, and LM27 epitope (PlantProbes, Leeds, UK, Table 1) for grass xylan hemicelluloses. The arabinogalactan proteins (AGPs) were detected by monoclonal antibodies—JIM13, JIM14, LM2 (PlantProbes, Leeds, UK, Table 1). The immunolabeling procedure was carried out according to Lenartowska et al. [42] with modifications. The sections on the glass slides were first blocked in 2% bovine serum albumin (BSA, Merck KGaA, Darmstadt, Germany, formerly Sigma, St. Louis, MO, USA) in PBS for 3 h and then incubated with primary monoclonal antibodies overnight, diluted 1:20, washed 3 times with PBS buffer and incubated with goat anti-Rat IgG (H+L) cross-adsorbed secondary antibody—diluted in 1:100 conjugated with Cy3 (A10522, Thermo Fisher Scientific, Waltham, MA, USA, excitation: 550 nm, emission: 570 nm) for 1.5 h. In the end, the sections were washed 3 times with PBS buffer and 4 times with deionized water. The samples were coated with Citifluor™ AF1 (Electron Microscopy Science, Hatfield, PA, USA), closed with cover glass, and protected by nail polish. Observations were carried out using a confocal microscope (Olympus FV1200, Tokyo, Japan).

#### 2.2.5. Masking of Xylans by Pectins

The detection process of masking xylans epitope LM27 by pectins was carried out according to Davies et al. [44] with modifications. The sections on the glass slides were first pre-treated with 2% pectinase (Merck KGaA, Darmstadt, Germany, formerly Sigma, St. Louis, MO, USA) in PBS buffer for 30 min, washed 3 times with PBS buffer. Then, the detection process of xylans with use of LM27 antibody, was carried out as described above. Some sections were carried out with control reaction using LM19 antibody.

## 3. Results

### 3.1. Colonization of Wheat Roots by Trichoderma spp.

**Figure 2:**
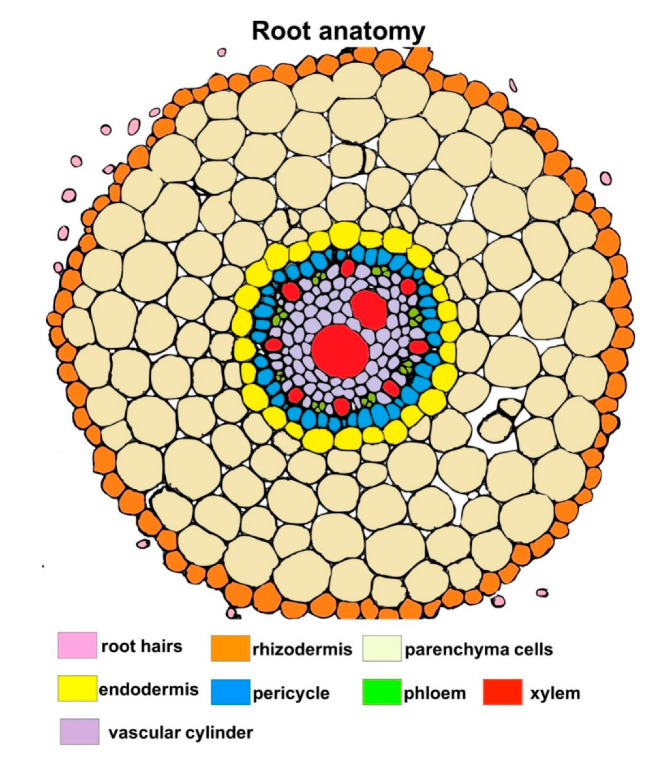
Schematic overview of wheat root anatomy.

**Figure 3:**
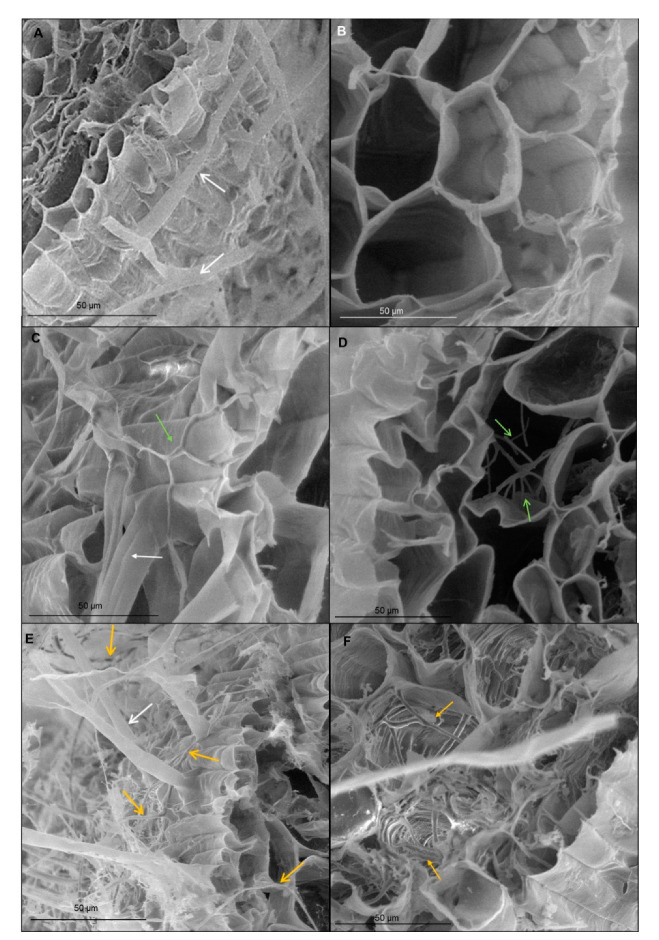
Micrographs of spring wheat seedlings (cv. Bombona) taken on scanning electron microscopy (SEM): (**A**,**B**)—control roots; (**C**,**D**)—roots treated with *T. atroviride* AN35, (**E**,**F**)—roots treated with *T. cremeum* AN392. White arrows indicate root hairs. Green arrow indicate hyphae of *T. atroviride* AN35. Yellow arrow indicate to hyphae of *T. cremeum* AN392.

**Figure 4:**
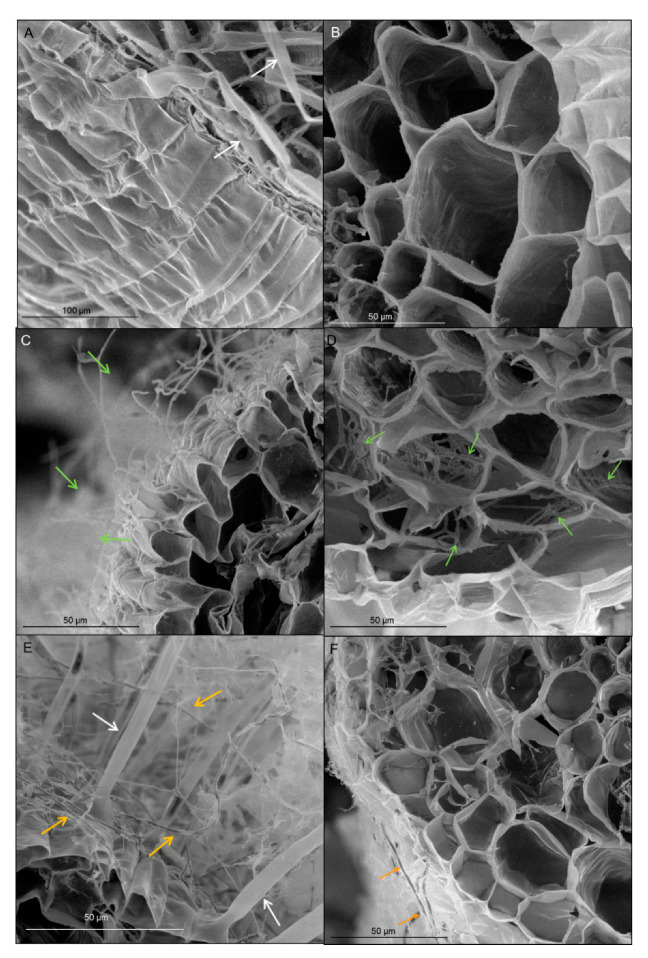
Micrographs of winter wheat seedlings (cv. Legenda) taken on scanning electron microscopy (SEM): (**A**,**B**)—control roots, (**C**,**D**)—roots treated with *T. atroviride* AN35, (**E**,**F**)—roots treated with *T. cremeum* AN392. White arrows indicate root hairs. Green arrow indicate hyphae of *T. atroviride* AN35. Yellow arrow indicate to hyphae of *T. cremeum* AN392.

**Figure 5:**
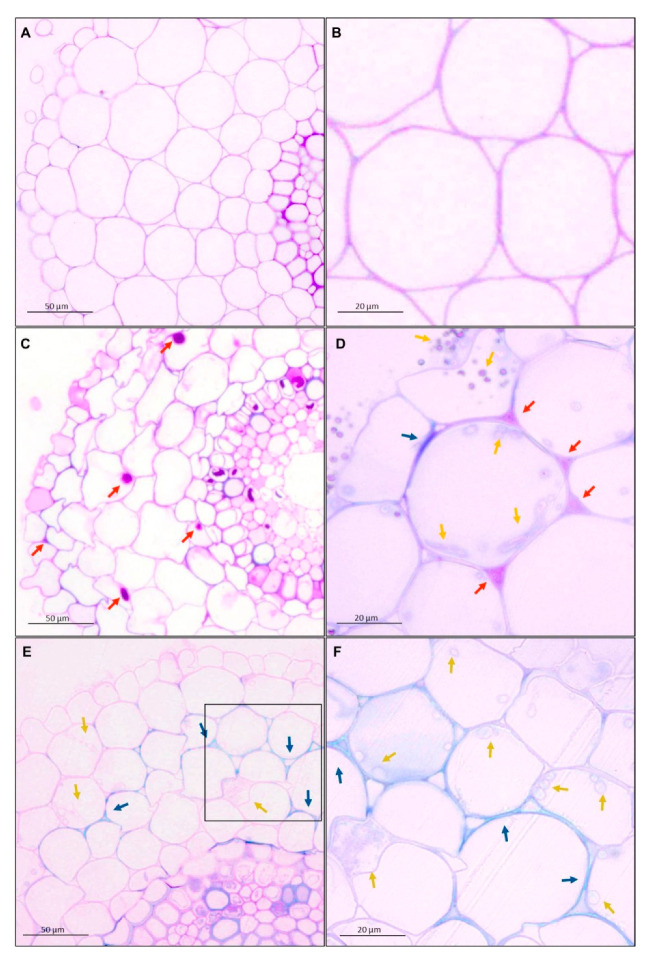
Micrographs of spring wheat seedlings (cv. Bombona) taken on light microscopy. The sections of roots were treated with toluidine blue solution: (**A**,**B**)—control roots, (**C**,**D**)—roots treated with *T. atroviride* AN35, (**E**,**F**)—roots treated with *T. cremeum* AN392. Red arrows indicate pectins. Blue arrows indicate lignins. Yellow arrows indicate hyphae of *T. atroviride* AN35 and*. cremeum* AN392.

**Figure 6:**
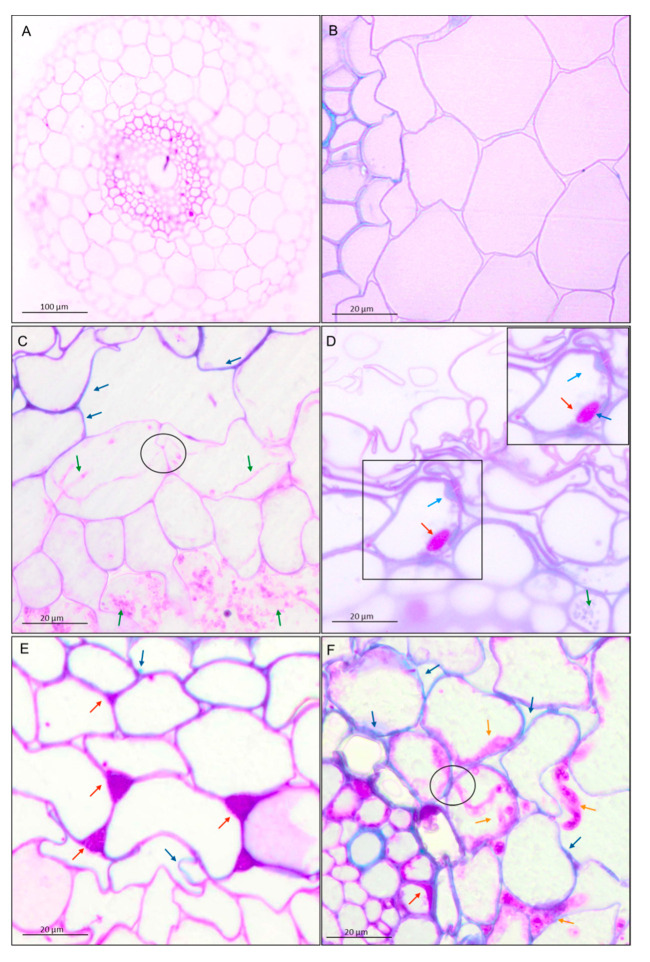
Micrographs of winter wheat seedlings (cv. Legenda) taken on light microscopy. The sections of roots were treated with toluidine blue solution: (**A**,**B**)—control roots, (**C**,**D**)—roots treated with *T. atroviride* AN35, (**E**,**F**)—roots treated with *T. cremeum* AN392. Red arrows indicate pectins. Blue arrows indicate lignins. Yellow arrows indicate hyphae of *T. atroviride* AN35 and *T. cremeum* AN392. Circles indicate a fungal hyphae growth from cell-to-cell by plant cell wall.

Transverse section of wheat root showed tissues: rhizodermis and root hairs, parenchyma cells, endodermis, pericycle and vascular bundle: xylem and phloem (Figure 2). Scanning electron microscopy and the light microscopy (where sections were dyed by toluidine blue) observations indicated that both *T. atroviride* AN35 and *T. cremeum*—AN392 can colonize the roots of both cultivars of wheat seedlings. Fungal hyphae growth on the surface of roots (Figure 3C and Figure 4C for *T. atroviride* AN35—green arrows and Figure 3E and Figure 4E for *T. cremeum* AN392—orange arrows). Fungal hyphae was also visible inside the root parenchyma cells (Figure 3D and Figure 4D for *T. atroviride* AN35 and Figure 3F and Figure 4F for *T. cremeum* AN392). The light microscopy observations revealed that hyphae growth in the lumen of parenchyma (Figure 5D and Figure 6C,D for *T. atroviride* AN35, Figure 5F and Figure 6F for *T. cremeum* AN392) and rhizodermis cells (Figure 5D) was seen. The hyphae were in the apoplast and grew from cell-to-cell, with barriers such as the plant cell walls (Figure 6C,F).

Observations of fungal hyphae were based on morphological differences between control and material treated with *Trichoderma*. In SEM, hyphae are visible, like thin, long, and divided structures, which are not present in the control root. On micrographs taken on light microscopy, sections of fungal hyphae are visible, oval or long curved structures, which are not present in the control.

### 3.2. Microscopy Observations of the Impact of Trichoderma spp. on Plant Cell Walls

#### 3.2.1. Pectins and Lignins

The shapes of cells in the control group were regular. Nevertheless, after interaction with both the fungi, the shapes of the cells became more irregular. Moreover, the deposition of the materials in the plant cell walls was different in the experimental group as compared to the deposition in the control group. The main component of the plant cell walls of wheat seedlings in the control group and experimental group were pectins. However, after interactions with *Trichoderma* spp., pectins appeared in intercellular spaces (red–purple color and red arrows, Figure 5D and Figure 6E). In the parenchyma cells, lignins were visible (blue color and blue arrows, Figure 5 and Figure 6). It was also observed that the cell walls were thickened by pectins (Figure 5C and Figure 6D) and lignins (Figure 6D).

#### 3.2.2. Immunolocalization of Pectins

**Figure 7:**
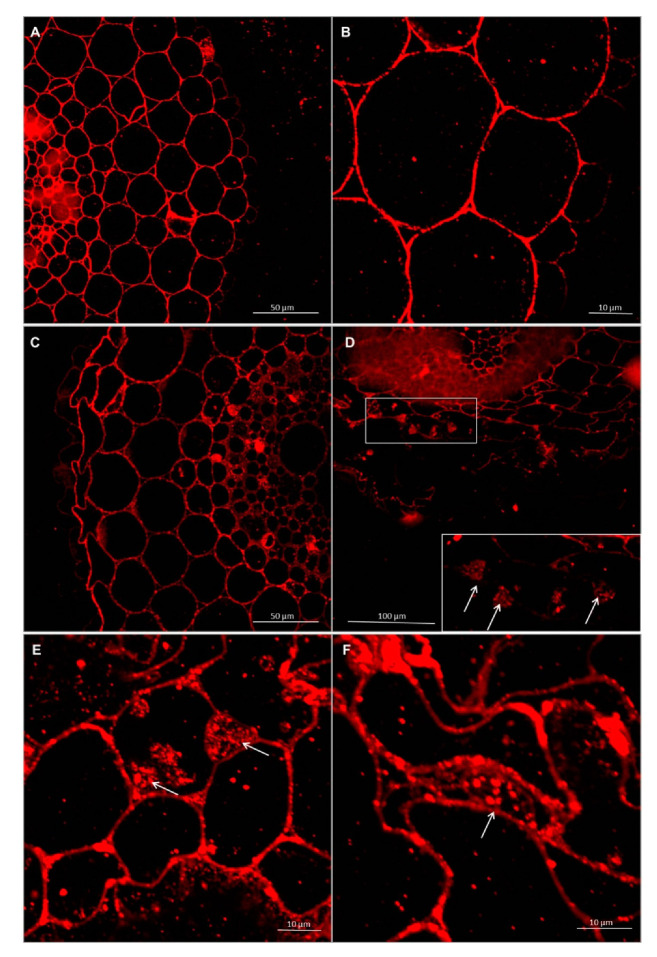
Immunolocalization of low methyl-esterified pectins LM19 epitope in roots of spring wheat (cv. Bombona): (**A**,**B**)—control roots, (**C**,**D**)—roots treated with *T. atroviride* AN35, (**E**,**F**)—roots treated with *T. cremeum* AN392. White arrows indicate pectins of LM19 epitope in intercellular spaces and cell wall thickening of the cells.

**Figure 8:**
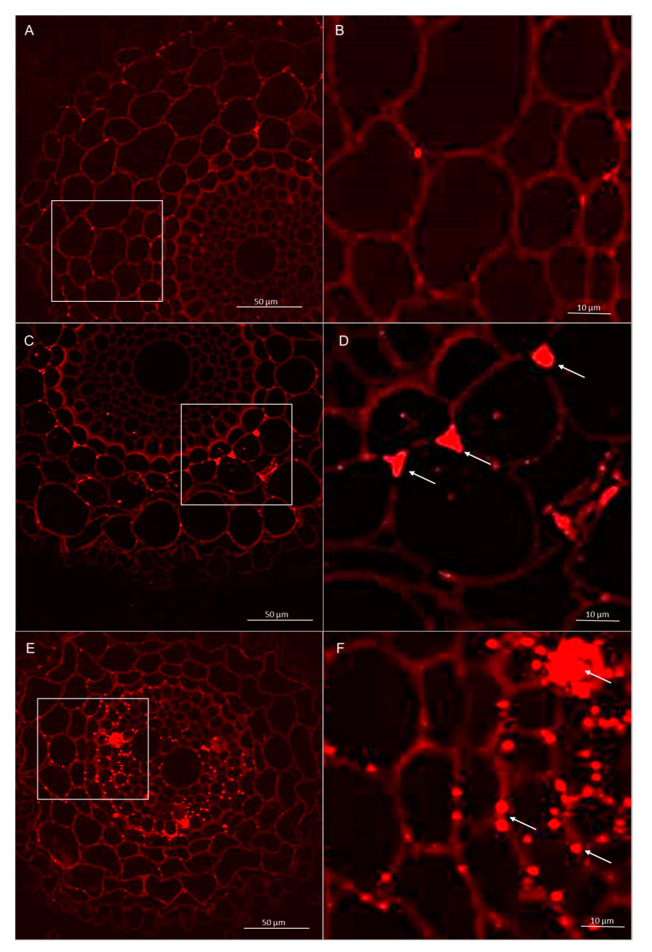
Immunolocalization of low methyl-esterified pectins LM19 epitope in roots of winter wheat (cv. Legenda): (**A**,**B**)—control roots, (**C**,**D**)—roots treated with *T. atroviride* AN35, (**E**,**F**)—roots treated with *T. cremeum* AN392. White arrows indicate pectins of LM19 epitope in intercellular spaces.

Immunolabeling via LM19 antibodies indicated that unesterified homogalacturonan was deposited in the cell walls of the control and experimental. It was also observed that the pectin epitope—LM19 was less abundant in the rhizodermis. In the material treated with *Trichoderma* spp., the accumulation of pectins was visible in the intercellular spaces, and this deposition contained unesterified homogalacturonan (Figure 7 and Figure 8). In cultivar Bombona after interaction with *T. cremeum* AN392, a cell wall thickening labeled by LM19 antibody was observed (Figure 7E).

#### 3.2.3. Immunolocalization of Hemicelluloses

**Figure 9:**
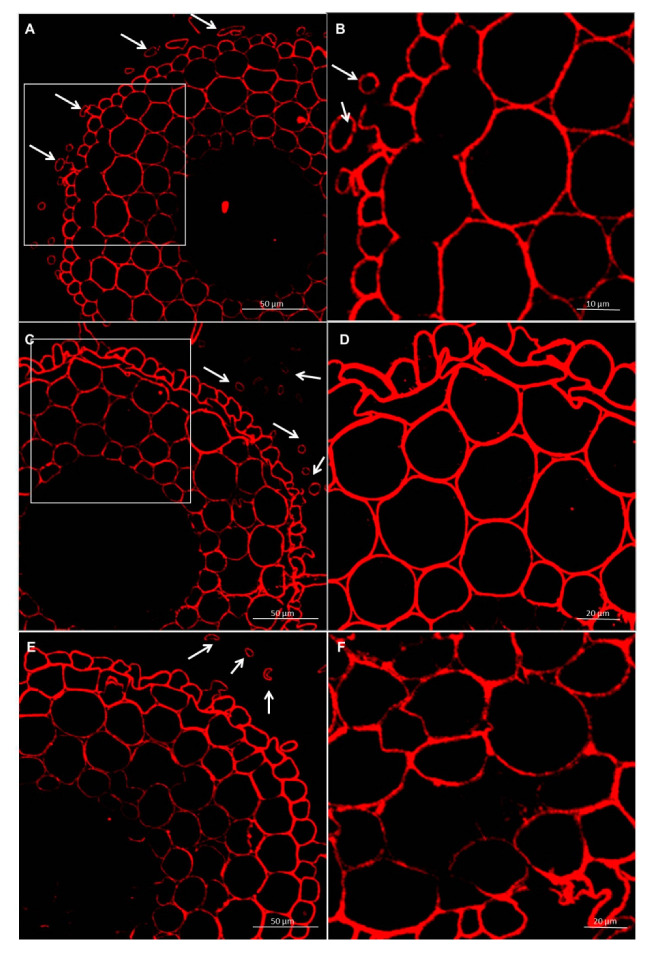
Immunolocalization of xylan LM27 epitope in roots of spring wheat (cv. Bombona): (**A**,**B**)—control roots, (**C**,**D**)—roots treated with *T. atroviride* AN35, (**E**,**F**)—roots treated with *T. cremeum* AN392. White arrows indicate root hairs.

**Figure 10:**
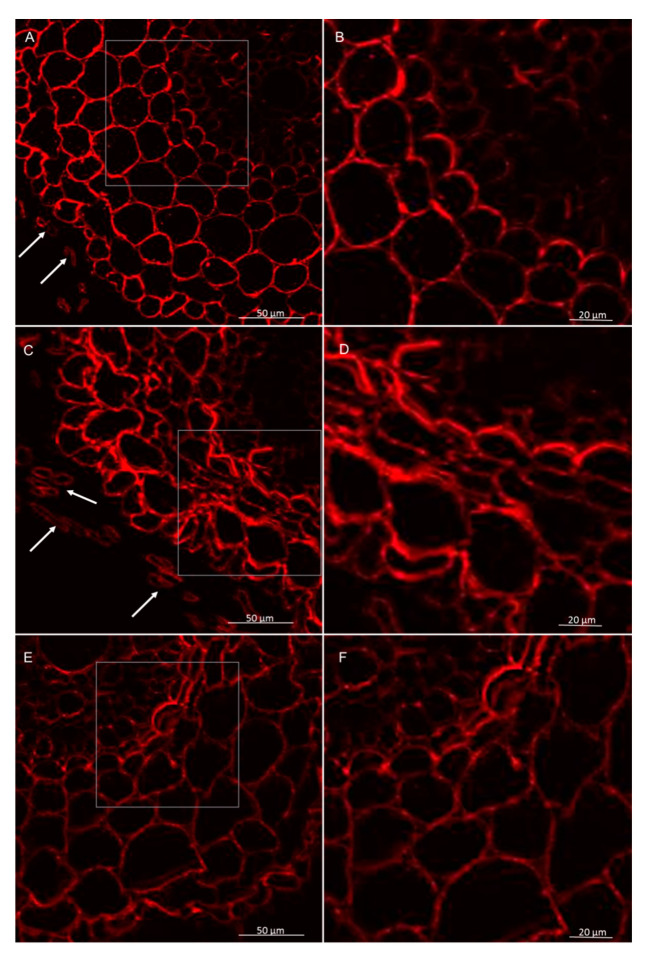
Immunolocalization of xylan LM27 epitope in roots of winter wheat (cv. Legenda): (**A**,**B**)—control roots, (**C**,**D**)—roots treated with *T. atroviride* AN35, (**E**,**F**)—roots treated with *T. cremeum* AN392. White arrows indicate root hairs.

**Figure 11:**
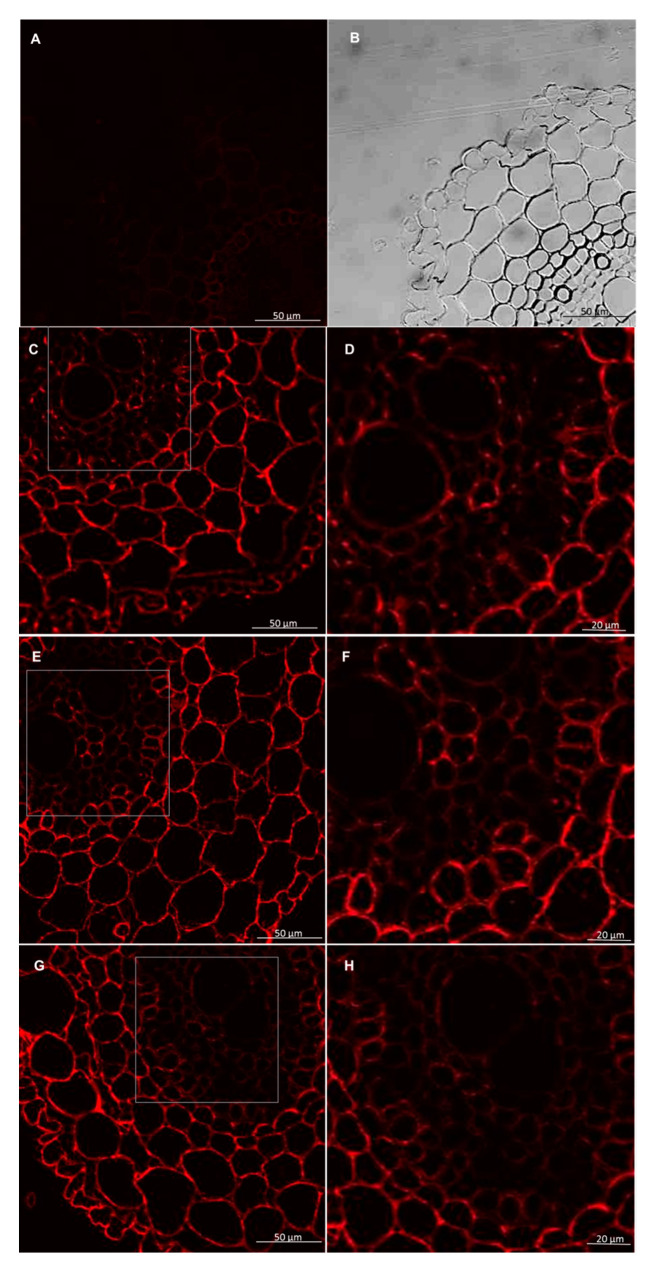
Immunolocalization of xylan LM27 epitope following treatment with pectinase in roots of spring wheat (cv. Bombona). Micrographs (**A**,**B**)—control reaction with using LM19 antibody. Micrographs (**C**–**H**)—reaction with using LM27 antibody: (**C**,**D**)—control roots, (**E**,**F**)—roots treated with *T. atroviride* AN35, (**G**,**H**)—roots treated with *T. cremeum* AN392.

**Figure 12:**
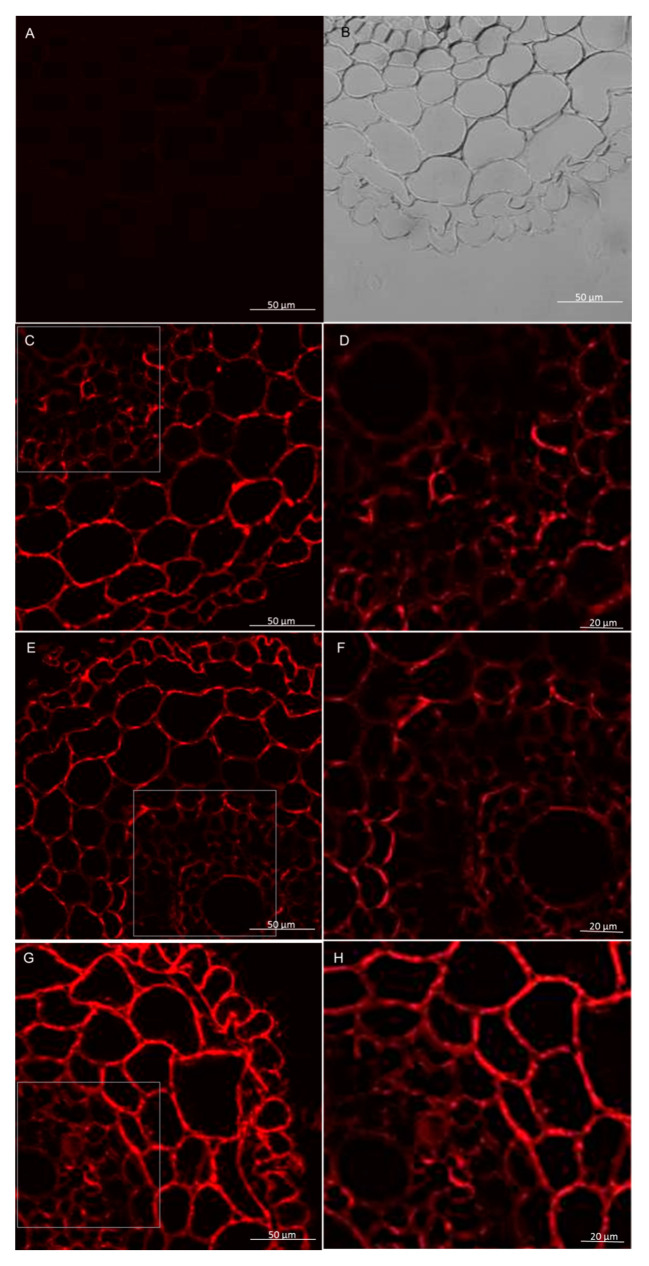
Immunolocalization of xylan LM27 epitope following treatment with pectinase in roots of winter wheat (cv. Legenda). Micrographs (**A**,**B**)—control reaction with using LM19 antibody. Micrographs (**C**–**H**)—reaction with using LM27 antibody: (**C**,**D**)—control roots, (**E**,**F**)—roots treated with *T. atroviride* AN35, (**G**,**H**)—roots treated with *T. cremeum* AN392.

Immunolabeling method using the LM27 antibody, specifically for grass xylans, showed that this fraction of hemicellulose in the roots of wheat seedling was mainly present in the root hairs, rhizodermis, and parenchyma cells (Figure 9 and Figure 10). Analysis of masking xylans LM27 epitope by pectins showed that after using of pectinase pectins LM19 epitope were removed from this samples (Figure 11A,B and Figure 12A,B). Nevertheless, analysis showed that xylans LM27 epitope was masked by pectins LM19 epitope in tissues: endodermis, pericycle, vascular cylinder, phloem, and xylem; however, in this tissue, xylans were less abundant (Figure 11 and Figure 12). Nevertheless, even after the interaction with both the *Trichoderma* spp., this xylan fraction remained unchanged.

#### 3.2.4. Immunolocalization of Arabinogalactan Proteins

**Figure 13:**
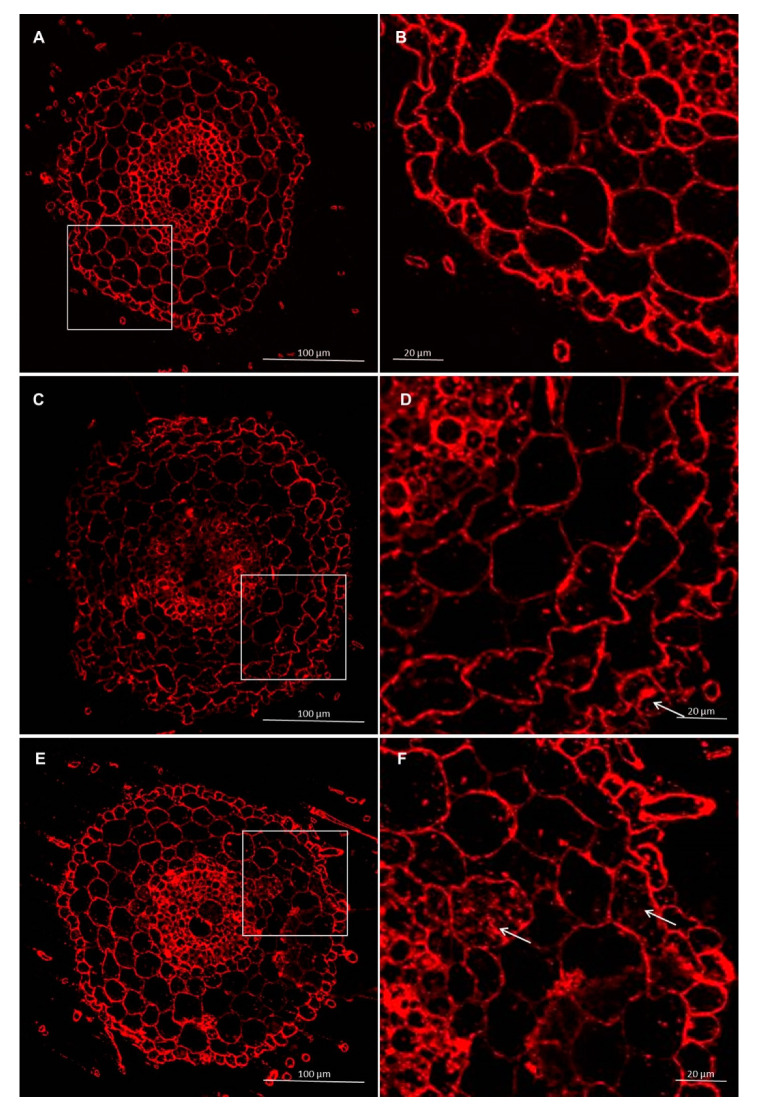
Immunolocalization of arabinogalactan proteins (AGPs) JIM13 epitope in roots of spring wheat (cv. Bombona): (**A**,**B**)—control roots, (**C**,**D**)—roots treated with *T. atroviride* AN35, (**E**,**F**)—roots treated with *T. cremeum* AN392. White arrows indicate arabinogalactan proteins (AGPs) JIM13 epitope inside of the parenchyma and rhizodermis cells.

**Figure 14:**
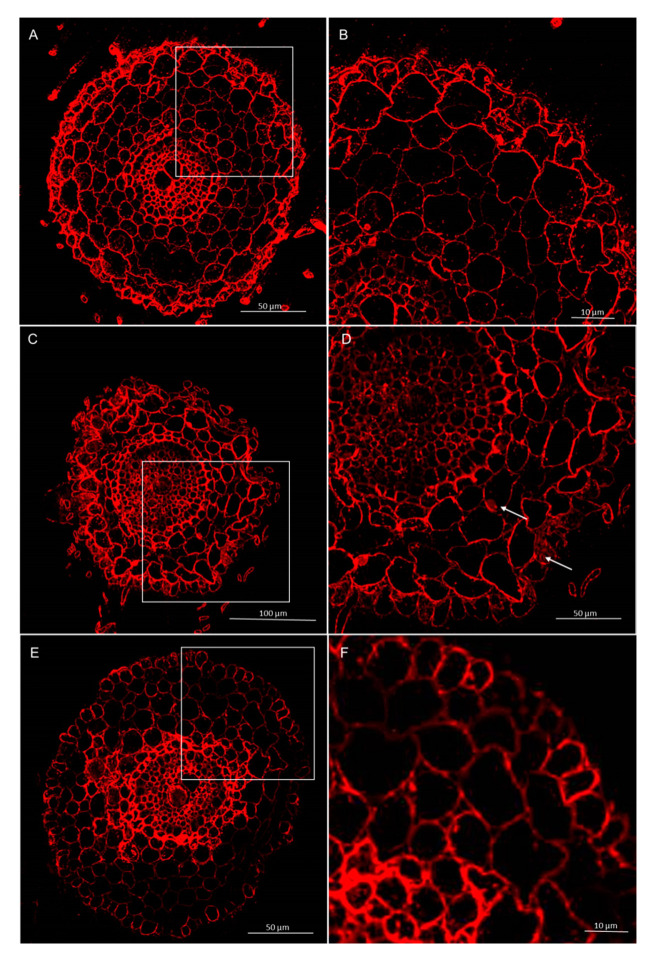
Immunolocalization of arabinogalactan proteins (AGPs) JIM13 epitope in roots of winter wheat (cv. Legenda): (**A**,**B**)—control roots, (**C**,**D**)—roots treated with *T. atroviride* AN35, (**E**,**F**)—roots treated with *T. cremeum* AN392. White arrows indicate AGPs JIM13 epitope inside of the parenchyma and rhizodermis cells.

**Figure 15:**
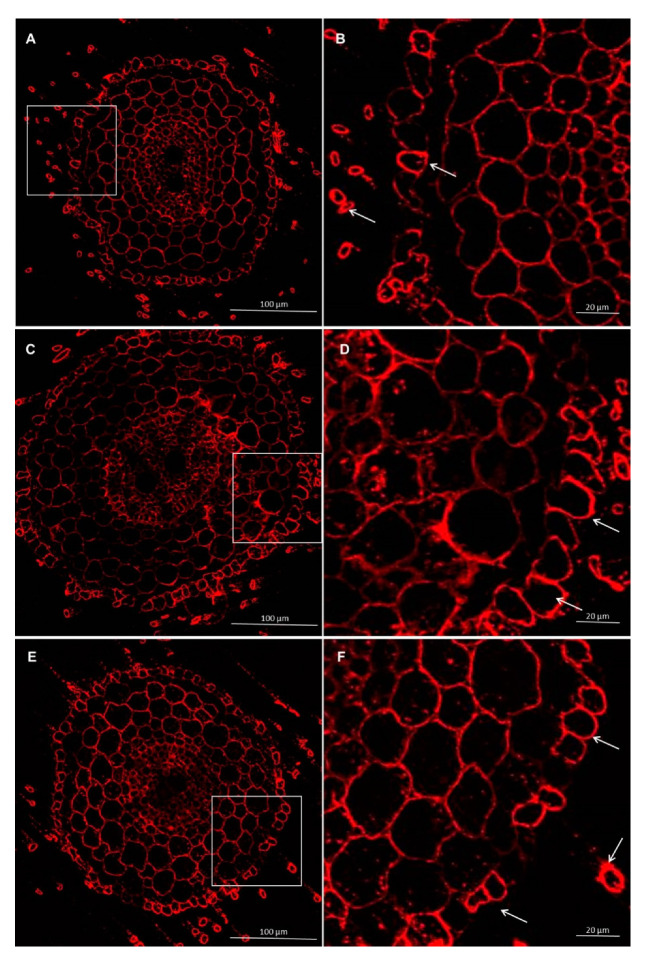
Immunolocalization of arabinogalactan proteins (AGPs) LM2 epitope in roots of spring wheat (cv. Bombona): (**A**,**B**)—control roots, (**C**,**D**)—roots treated with *T. atroviride* AN35, (**E**,**F**)—roots treated with *T. cremeum* AN392. White arrows indicate AGPs LM2 epitope in trichoblasts and root hairs.

**Figure 16:**
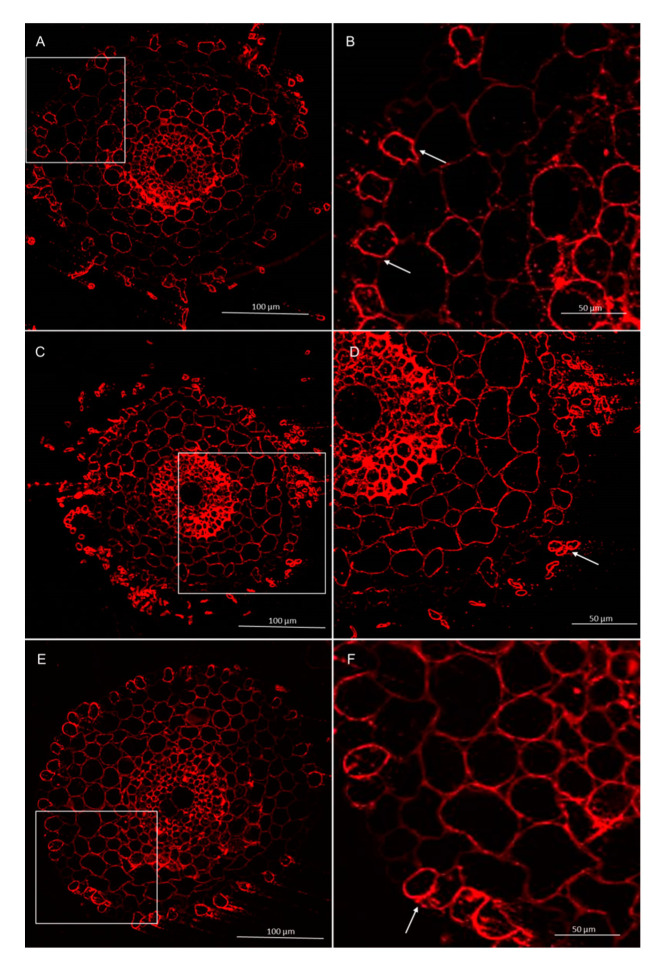
Immunolocalization of arabinogalactan proteins (AGPs) LM2 epitope in roots of winter wheat (cv. Legenda): (**A**,**B**)—control roots, (**C**,**D**)—roots treated with *T. atroviride* AN35, (**E**,**F**)—roots treated with *T. cremeum* AN392. White arrows indicate LM2 epitope in trichoblasts and root hairs.

**Figure 17:**
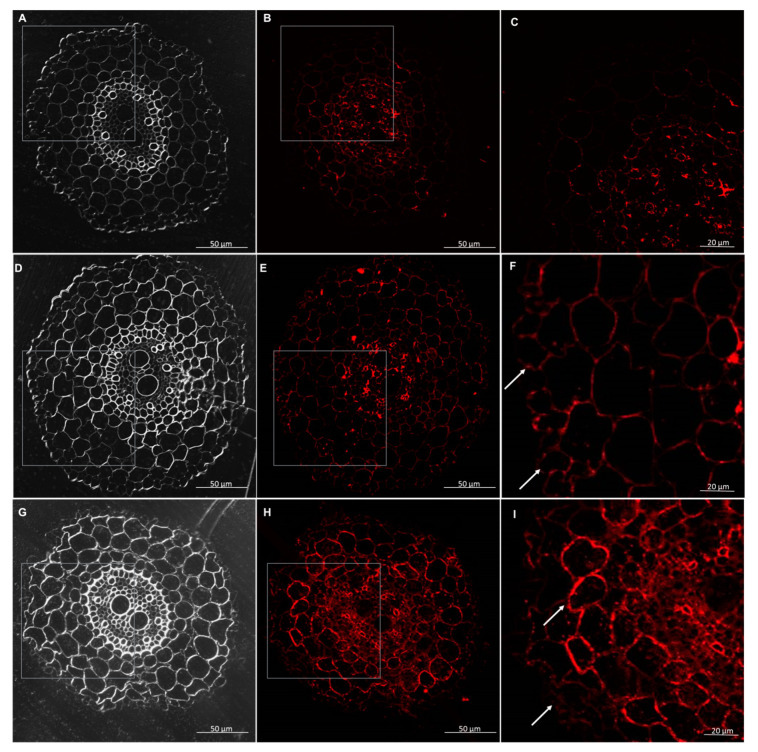
Immunolocalization of arabinogalactan proteins (AGPs) JIM14 epitope in roots of spring wheat (cv. Bombona): (**A**–**C**)—control roots, (**D**–**F**)—roots treated with *T. atroviride* AN35, (**G**–**I**)—roots treated with *T. cremeum* AN392. White arrows indicate JIM14 epitope in parenchyma and rhizodermis cells.

**Figure 18:**
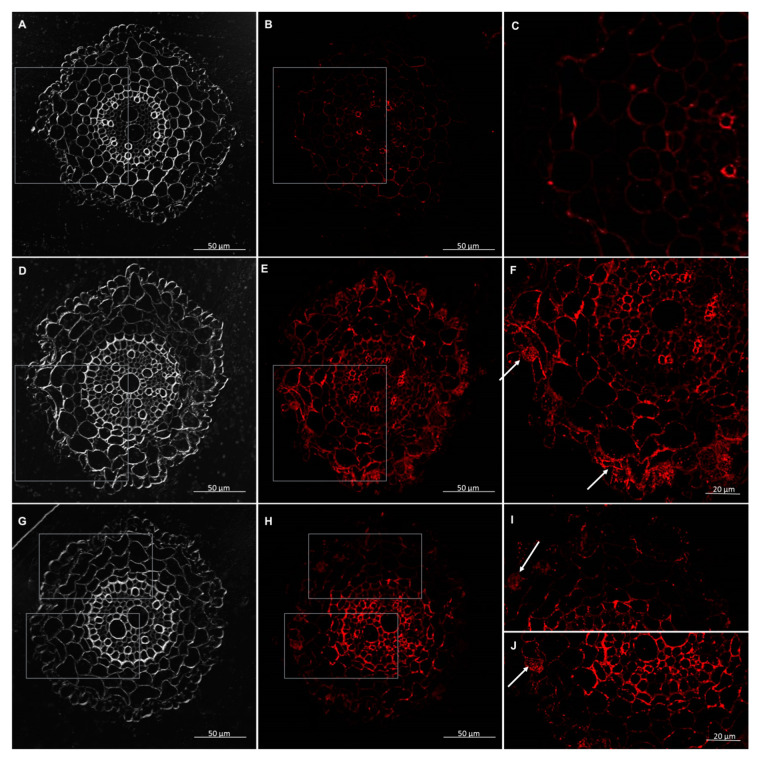
Immunolocalization of arabinogalactan proteins (AGPs) JIM14 epitope in roots of winter wheat (cv. Legenda): (**A**–**C**)—control roots, (**D**–**F**)—roots treated with *T. atroviride* AN35, (**G**–**J**)—roots treated with *T. cremeum* AN392. White arrows indicate accumulation of JIM14 epitope in rhizodermis cells.

The AGPs were detected by monoclonal antibodies—JIM13, JIM14, and LM2. The results demonstrated that in the wheat seedlings, the AGP epitope—JIM13 occurred in the cell walls, in the root hair, and each tissue in the control conditions and after interactions with *T. atroviride* AN35 and *T. cremeum* AN392. The AGPs epitope, JIM13, was present inside of the parenchyma and rhizodermis cells in all variants (Figure 13 and Figure 14). The AGP epitope, LM2, was present in trichoblasts and root hair in parenchyma cells without the first layer under the rhizodermis and stele tissues in the control and experimental groups (Figure 15 and Figure 16). The AGP epitope, JIM14, in the control condition was mostly visible in xylem. However, after interaction with both the *Trichoderma* spp., localization of this epitope was modified. It was strongly visible in parenchyma cells, especially in the cell walls of a few layers of parenchyma cells under the rhizodermis. The AGPs of this epitope were also detected in the cell walls of the rhizodermis and could be accumulated in the cytoplasm of the rhizodermis cells (Figure 17 and Figure 18).

## 4. Discussion

The results demonstrated that both *T. atroviride* AN35 and *T. cremeum* AN392 colonize the roots of wheat seedlings. The microscopy analysis showed that the hyphae of both the *Trichoderma* grew on the outer layer of the roots of the wheat seedlings. This behavior was also observed in *T. atroviride* during the colonization of *Arabidopsis* roots [45]. The present study has also showed that *T. atroviride* AN35 and *T. cremeum* AN392 grew in the apoplast, in intercellular spaces, and into the parenchyma cells of wheat roots. The same behavior was observed for other *Trichoderma* strains in their interaction with different plants. For example, it was shown that, *Trichoderma harzianum* CECT 2413 grew into the roots of tomato [41], and also colonized olive roots [46].

This study revealed that the composition of the plant cell walls of wheat seedling roots changed after their interaction with *T. atroviride* AN35 and *T. cremeum* AN392. Yedida et al. [47] showed that electronically dense material was found in intercellular spaces of cucumber roots after their interaction with *Trichoderma*. Results obtained in this study have shown that pectins reorganized themselves after interacting with *T. atroviride* AN35 and *T. cremeum* AN392. Pectins appeared in intercellular spaces, and the immunocytochemistry reaction showed that it could be an unesterified homogalacturonan. Deesterified pectins (JIM5 epitope) were detected in the cell wall of *Allium porrum* in interaction with the arbuscular mycorrhizal fungus (AMF), *Glomus versiforme* [48], and in *Corylus avellana*, treated with *Tuber magnatum* [49]. It has been documented that the esterification of pectins also occurs while plants interact with pathogenic fungi. The lower activity level of pectin methyl esterases (PME), response for modulation, and the degree and patterns of methyl esterification of pectins caused a higher degree of methyl esterification of pectins in the cell walls of transgenic durum wheat (with a higher expression of pectin methyl esterase inhibitor (PMEI)—antagonist of pectin methyl esterases (PME), thereby increasing the resistance of these plants to pathogens like *B. sorokiniana* or *Fusarium graminearum*. A higher degree of methyl esterification caused more resistance to fungal endopolygalacturonases (PGs) [50]. It was also shown that in resistant wheat plants, the activity of the *WheatPME1* gene was downregulated in the resistance line to Fusarium Head Blight (FHB) and induced in the susceptibility line. During the early stages of *Fusarium* infection in wheat plants, the level of methyl esterification was lower [51]. Moreover, pectin deesterification can be involved in a plant’s defense strategy, in the first steps of pathogen infection [52] as pectins are a source of oligogalacturonides (OG)—important endogenous molecules, which are known to play a crucial role in the plant’s defense response to pathogens. Oligogalacturonides are short fragments of homogalacturonan caused by microbial and endogenous polygalacturonidases [53]. The pattern of methyl esterification is critical for the action of these enzymes. Oligogalacturonides need to be deesterified to elicit a defense response [54]. In strawberry plants, oligogalacturonides with a low degree of methyl esterification were correlated with the defense response to *Botrytis cinerea* [54]. Nevertheless, it was shown that acetylation of OG reduced the haustoria of *Blumeria graminis* wheat leaves [55]. Taking into account that the currently studied strains *Trichoderma*—mycoparasitic *T. atroviride* and saprotrophic *T. cremeum* are not pathogens of plants, it can be assumed that reorganization of pectins included low methyl-esterified pectins in cell walls of wheat roots after interaction with *Trichoderma* strains is a manifestation of symbiotic interactions.

The results obtained in this research showed that in the parenchyma cells of wheat roots, lignins are formed as a result of the interaction with both the *Trichoderma*. Deposition of these molecules in the plant cell walls makes them more resistant to cell wall degrading enzymes (CWDEs), spread the toxins into the host [32], and increases their resistance to pathogens, like the increased resistance of cotton plants to *Verticillium dahliae* [56] and Eucalyptus plants to *Mycosphaerella* fungi [57]. The data indicate that following the interaction of plants with pathogens, the genes responsible for lignin biosynthesis are upregulated [58,59]. Chacón et al. [41] demonstrated that the colonization of tomato roots by *T. harzianum* leads to modification in the cell walls in the form of lignin depositions. Lignins were also present in cucumber shoots after their interaction with *T. atroviride* TR25 [60]. We suppose that lignin deposition in wheat roots after treated with tested *Trichoderma* strains showed positive impact on this plants. After interaction with *T. atroviride* AN35 and *T. cremeum* AN392 deposited lignins in wheat plant cell wall constitutes a physical barrier to pathogen invasion into wheat roots. It was also shown that inoculation of cucumber roots by *T. atroviride* TR25 can trigger on callose deposition in shoots of this plant [60]. The data indicates that callose was synthesized in plant cell walls after pathogen infection, it is believed that depositions of callose is physically protect plants by pathogen invasion [61]. On the other side, Nishimura et al. [62] showed that in *Arabidopsis* mutant: *powdery mildew resistant 4* (*pmr4*), lacking pathogen-induced callose, despite this, is resistant to pathogens. They showed that in plants mutants after infection by pathogen, genes connected with on salicylic acid (SA) pathway were up-regulated. They conclude that callose synthesis affected negatively on salicylic acid (SA) pathway [62], which take a role in systemic acquired resistance (SAR) [63]. They also showed that in mutant plants papilla like structures were appear in plant cell walls after pathogen infection, but they are lacking in callose deposition [62].

The microscopy observations in this study revealed that in the control wheat roots and the wheat roots exposed to both *Trichoderma* hemicelluloses, the xylans epitope—LM27 in roots are present in the rhizodermis and parenchyma cells. However, data show that hemicelluloses can be masked by pectins [64]. Microscopy observations in this study showed that in wheat seedlings xylans LM27 epitope can be masked by pectins LM19 epitope in tissues like: endodermis, pericycle, vascular cylinder, phloem, and xylem. Nevertheless, in these tissues, xylans are less abundant. Hemicelluloses can play a role in the plant’s defense strategy to pathogens. Data show that the expression of gene encoding enzymes responsible for xylan biosynthesis makes plants more resistant to infection by *B. graminis* [31]. Changes in acetylation of xylans and pectins also influence a plant’s resistance to pathogens. Transformation of *Arabidopsis* and *Brachypodium distachyon* to express acetylesterases from *Aspergillus nidulans* increased the resistance of these plants to the fungal pathogens *Botrytis cinerea* and *Bipolaris sorokiniana* [65]. Data indicate that transgenic durum wheat produces xylanase inhibitors to counteract microbial xylanases. Expression of these enzymes in plants increased their resistance to *F. graminearum,* but do not to *B. sorokiniana* [66]. However, the results obtained in this research did not reveal changes in the reorganization of the xylan LM27 epitope after interaction with *Trichoderma*. With this in mind, it is believed that xylans epitope LM27 did not take a role in plant cell wall interaction with beneficial fungi of *Trichoderma,* but this does not exclude the involvement of other hemicelluloses in these interactions, which, however, would have to be confirmed in further analyzes.

AGPs play diverse roles in a plant’s development, growth, defense strategy, and also in the colonization of plant roots by different microorganisms [37]. Each of the recognized epitope of the AGPs by the antibody is located in a specific tissue on plant organ [35]. The results obtained in this research showed that, in wheat seedlings, after their interaction with *Trichoderma*, the AGP epitope JIM13 occurred in all types of root tissues in the control group and experimental group. Nevertheless, [67] showed that after the interaction of *Alnus* spp. with nitrogen-fixing symbiotic *Frankia*, the AGP epitope, JIM13, appeared in a lot of in the symbiotic interface at the membrane cell wall of the border cells. The AGP JIM13 epitope was also detected in the cytoplasm and vacuole at a mature stage of infection. The subcellular localization of JIM13 suggests that these AGPs can play a role in the proliferation of *Frankia* in the nodule. Berry et al. [67] also demonstrated that AGPs can interact with partially methyl-esterified epitopes of homogalacturonan, epitope JIM7, which were found in colocalization with the AGP epitope JIM4. The result obtained in our study indicated that in wheat roots, the AGP epitope, JIM14, can play a role in response to *Trichoderma*. In roots treated with fungi, proteins of this epitope abundantly occurred in the cell walls of parenchyma and of rhizodermis and cytoplasm of rhizodermis. The data showed that other AGPs can play a role in the interaction of plants with pathogens or symbiotic microorganisms. A study that focused on the interaction between mycorrhizal AM Glomus fungi and *Medicago truncatula* showed that in cells with mycorrhiza abundant transcripts of AGPs were found [68]. However, it was found that *Arabidopsis* with a mutation in the AGP17 gene was not transformed by *Agrobacterium tumefaciens*. Treated wild plants with Yariv reagent also demonstrated a reduced frequency of transformation by *Agrobacterium*. Nevertheless, recovering the AGP 17 gene from wild plants restored the mutant’s ability to transform [69]. Moreover, the microscopic observations obtained in this research revealed that localization of the tested AGPs, such as JIM13 (in all tissues), LM2 (in trichoblasts and root hairs, and parenchyma cells without the first layer under the rhizodermis), and JIM14 (mostly in xylem) in the control group showed localization of AGP epitopes similar to that in barley roots [35], and can play a role in wheat root development. Thus, the data indicate that AGPs can act as a part of plant response to both symbiotic and pathogenic microorganisms. Here, it can be assumed that wheat AGP epitope JIM14 was involved in establishing of symbiotic interactions with fungi of *T. atroviride* AN35 a and *T. cremeum* AN392.

The obtained results showed that both the tested *Trichoderma* species can colonize the roots of wheat seedlings. This interaction triggered changes in plant cell walls in the form of lignin depositions and rearrangement of pectins, including low methyl-esterified pectins. This research also showed that the AGP epitope JIM14 can participate in the wheat seedling-*Trichoderma* interactions. Regarding previous reports, it is concluded that the changes in plant cell walls observed here in wheat seedlings as a result of interaction with *T. atroviride* AN35 and *T. cremeum* AN392 and their potential to colonize the root endosphere of wheat may indicate ability of these strains to form symbiotic interactions with wheat. Bearing in mind the non-pathogenicity of these fungi towards plants, which has been numerously documented previously [3,4], it can be assumed that the tested strains may have a beneficial effect on wheat plants. Moreover, these and their previously documented antagonistic abilities against *Fusarium* species, including the potential to produce antifungal volatile metabolites and lytic enzymes [16,23,70,71], may predispose these *Trichoderma* strains to be used as potential biological control agents. Nevertheless, further experiments with the use this two *Trichoderma* strains in field conditions, extended with the analysis of the impact of both fungi on the wheat hormonal metabolism, as suggested by research of Nishimura et al. [63], are required to approved the beneficial effects of *T. atroviride* AN35 and *T. cremeum* AN392 on wheat plants and their potential for use in biological control.

## Figures and Tables

**Table 1:** Antibodies used in detection of plant cell wall molecules in wheat roots.

mAbs	Recognized Epitope
LM19	un-esterified homogalacturonan [43]
LM27	Grass xylan [43]
JIM13	β-D-GlcA-(1,3)-α-D-GalA-(1,2)-α-L-Rha [43]
JIM14	unknown structure of epitope [43]
LM2	carbohydrate epitope containing b-linked glucuronic acid [43]

## References

[1] Food and Agriculture Organization of the United Nations, FAO. http://www.fao.org/worldfoodsituation/csdb/en/.

[2] Figueroa M., Hammond-Kosack K.E., Solomon P.S. (2018). A review of wheat diseases—A field perspective. Mol. Plant Pathol..

[3] Harman G.E., Howell C.R., Viterbo A., Chet I., Lorito M. (2004). *Trichoderma* species—Opportunistic, avirulent plant symbionts. Nat. Rev. Microbiol..

[4] Druzhinina I.S., Seidl-Seiboth V., Herrera-Estrella A., Horowitz B.A., Kenerley C.M., Monte E., Mukherjee P.K., Zeilinger S., Grigoriev I.F., Kubicek C.P. (2011). *Trichoderma*: The genomics of opportunistic success. Nat. Rev. Microbiol..

[5] Mukherjee P.K., Horwitz B.A., Herrera-Estrella A., Schmoll M., Kenerley C.M. (2013). *Trichoderma* research in the genome era. Annu. Rev. Phytopathol..

[6] Djonović S., Vargas W.A., Kolomiets M.V., Horndeski M., Wiest A., Kenerley C.M. (2007). A proteinaceous elicitor Sm1 from the beneficial fungus *Trichoderma virens* is required for induced systemie resistance in maize. Plant Physiol..

[7] Shoresh M., Harman G.E. (2008). The molecular basis of shoots responses of maize seedlings to *Trichoderma harzianum* T22 inoculation of the root: A proteomic approach. Plant Physiol..

[8] Shoresh M., Yedidia I., Chet I. (2005). Involvement of jasmonie acid/ethylene signaling pathway in the systemie resistance induced in cucumber by *Trichoderma asperellum* T203. Phytopathology.

[9] Viterbo A., Harel M., Horowitz B.A., Chet I., Mukherjee P.K. (2005). *Trichoderma* mitogen—Activated protein kinase signaling is involved in induction of plant systemic resistance. Appl. Environ. Microbiol..

[10] Djonović S., Pozo M.J., Dangott L.J., Howell C.R., Kenerley C.M. (2006). Sm1, a proteinaceous elicitor secreted by the biocontrol fungi *Trichoderma virens* induces plant defense responses and systemic resistance. Mol. Plant Microbe Interact..

[11] Nawrocka J., Małolepsza U. (2013). Diversity in plant systemic resistance induced by *Trichoderma*. Biol. Control..

[12] Bortman Y., Kapuganti J.G., Viterbo A. (2010). Trichoderma. Curr. Biol..

[13] Hermosa R., Viterbo A., Chet I., Monte E. (2012). Plant—Beneficial effects of *Trichoderma* and of its genes. Microbiology.

[14] Verma M., Brar S.K., Tyagi R.D., Surampalli R.Y., Valéro J.R. (2007). Antagonist fungi, *Trichoderma* spp.: Panoply of biological control. Biochem. Eng. J..

[15] Błaszczyk L., Siwulski M., Sobieralski K., Lisiecka J., Jędryczka M. (2014). *Trichoderma* spp.—Application and prospects for use in organic farming and industry. J. Plant Prot. Res..

[16] Błaszczyk L., Basińska-Barczak A., Ćwiek-Kupczyńska H., Gromadzka K., Popiel D., Stępień Ł. (2017). Suppressive Effect of *Trichoderma* spp. on toxigenic *Fusarium* species. Pol. J. Microbiol..

[17] Vinale F., Sivasithamparam K., Ghisalberti E.L., Marra R., Barbetti M.J., Li H., Woo S.L., Lorito M. (2008). A novel role for *Trichoderma* secondary metabolites in the interactions with plants. Physiol. Mol. Plant Pathol..

[18] El-Hasan A., Buchenauer H. (2009). Actions of 6-Pentyl-alpha-pyrone in Controlling Seedling Blight Incited by *Fusarium moniliforme* and Inducing Defence Responses in Maize. J. Phytopathol..

[19] Scarselletti R., Faull J.L. (1994). In vitro activity of 6-pentyl-a-pyrone, a metabolite of *Trichoderma harzianum*, in the inhibition of *Rhizoctonia solani* and *Fusarium oxysporum* f. sp. *lycopersici*. Mycol. Res..

[20] Worasatit N., Sivasithamparam K., Ghisalberti E.L., Rowland C. (1994). Variation in pyrone production, lytic enzymes and control of rhizoctonia root rot of wheat among single—spore isolates of *Trichoderma koningii*. Mycol. Res..

[21] Poole P.R., Ward B.G., Whitaker G. (1998). The effects of topical treatment with 6-pentyl-2-pyrone and structural analogues on stem and postharvest rots in kiwifruit due to *Botrytis cinerea*. J. Sci. Food Agric..

[22] Tarus P.K., Langat-Thoruwa C.C., Wanyonyi A.W., Chhabra S.C. (2003). Bioactive metabolites from *Trichoderma harzianum* and *Trichoderma longibrachiatum*. Bull. Chem. Soc. Ethiopia.

[23] Jeleń H., Błaszczyk L., Chełkowski J., Rogowicz K., Strakowska J. (2014). Formation of 6-n-pentyl-2H- pyran-2-one (6-PAP) and other volatiles by different *Trichoderma* species. Mycol. Prog..

[24] Błaszczyk L., Strakowska J., Chełkowski J., Gąbka-Buszek A., Kaczmarek J. (2016). *Trichoderma* species occurring on wood with decay symptoms in mountain forests in Central Europe: Genetic and enzymatic characterization. J. Appl. Genet..

[25] Keegstra K. (2010). Plant cell walls. Plant Physiol..

[26] Carpita N.C., Gibeaut D.M. (1993). Structural models of primary cell walls in flowering plants: Consistency of molecular structure with the physical properties of the walls during growth. Plant J..

[27] Marty P., Jouan B., Bertheau Y., Vian B., Goldberg R. (1997). Charge density in stem cell walls of *Solanum tuberosum* genotypes and susceptibility to blackleg. Phytochemistry.

[28] Boudart G., Lafitte C., Barthe J.P., Frasez D., Esquerré-Tugayé M.-T. (1998). Differential elicitation of defense responses by pectic fragments in bean seedlings. Planta.

[29] Ferrari S., Galletti R., Pontiggia D., Manfredini C., Lionetti V., Bellincampi D., Cervone F., de Lorenzo G. (2008). Transgenic expression of a fungal endopolygalacturonase increases plant resistance to pathogens and reduces auxin sensitivity. Plant Physiol..

[30] De Lorenzo G., Cervone F., Bellincampi D., Caprari C., Clark A.J., Desiderio A., Devoto A., Forrest R., Leckie F., Nuss L. (1994). Polygalacturonase, PGIP and oligogalacturonides in cell-cell communication. Biochem. Soc. Trans..

[31] Chowdhury J., Lück S., Rajaraman J., Douchkov D., Shirley N.J., Schwerdt J.G., Schweizer P., Fincher G.B., Burton R.A., Little A. (2017). Altered Expression of Genes Implicated in Xylan Biosynthesis Affects Penetration Resistance against Powdery Mildew. Front. Plant Sci..

[32] Sattler S.E., Funnell-Harris D.L. (2013). Modifying lignin to improve bioenergy feedstocks: Strengthening the barrier against pathogens?. Front. Plant Sci..

[33] Collinge D.B. (2009). Cell wall appositions: The first line of defence. J. Exp. Bot..

[34] Bhuiyan N.H., Selvaraj G., Wei Y., King J. (2009). Role of lignification in plant defense. Plant Signal. Behav..

[35] Marzec M., Szarejko I., Melzer M. (2015). Arabinogalactan proteins are involved in root hair development in barley. J. Exp. Bot..

[36] Lamport D.T.A., Kieliszewski M.J., Showalter A.M. (2006). Salt stress upregulates periplasmic arabinogalactan proteins: Using salt stress to analyse AGP function. New Phytol..

[37] Nguema-Ona E., Vicré-Gibouin M., Cannesan M.-A., Driouich A. (2013). Arabinogalactan proteins in root—Micro be interactions. Trends Plant Sci..

[38] Lee K.J., Sakata Y., Mau S.-L., Pettolino F., Bacic A., Quatrano R.S., Knight C.D., Knox J.P. (2005). Arabinogalactan proteins are required for apical cell extension in the moss *Physcomitrella patens*. Plant Cell.

[39] Błaszczyk L., Popiel D., Chełkowski J., Koczyk G., Samuels G.J., Sobieralski K., Siwulski M. (2011). Species diversity of *Trichoderma* in Poland. J. Appl. Genet..

[40] Marecik R., Błaszczyk L., Biegańska-Marecik R., Piotrowska-Cyplik A. (2018). Screening and Identification of *Trichoderma* Strains Isolated from Natural Habitats with Potential to Cellulose and Xylan Degrading Enzymes Production. Pol. J. Microbiol..

[41] Chacón M.R., Rodriguez-Galán O., Benitez T., Sousa S., Rey M., Llobell A., Delgado-Jarana J. (2007). Microscopic and transcriptome analyses of early colonization of tomato roots by *Trichoderma harzianum*. Int. Microbiol..

[42] Lenartowska M., Rodríguez-García M.I., Bernarska E. (2001). Immunocytochemical localization of esterified and unesterified pectins in unpollinated and pollinated styles of *Petunia Hybrid*. Hort. Planta.

[43] PlantProbes. http://www.plantprobes.net/index.php.

[44] Davies L.J., Lilley C.J., Paul Knox J., Urwin P.E. (2012). Syncytia formed by adult female *Heterodera schachtii* in *Arabidopsis thaliana* roots have a distinct cell wall molecular architecture. New Phytol..

[45] Salas-Marina M.A., Silva-Flores M.A., Uresti-Rivera E.E., Castro-Longoria E., Herrera-Estrella A., Casas-Flores S. (2011). Colonization of *Arabidopsis* roots by *Trichoderma atroviride* promotes growth and enhances systemic disease resistance through jasmonic acid/ethylene and salicylic acid pathways. Eur. J. Plant Pathol..

[46] Ruano-Rosa D., Prieto P., Rincón A.M., Gómez-Rodríguez M.V., Valderrama R., Barroso J.B., Mercado-Blanco J. (2016). Fate of *Trichoderma harzianum* in the olive rhizosphere: Time course of the root colonization process and interaction with the fungal pathogen *Verticillium dahliae*. BioControl.

[47] Yedida I., Benhamou N., Chet I. (1999). Induction of Defense Responses in Cucumber Plants (*Cucumis sativus* L.) by the Biocontrol Agent *Trichoderma harzianum*. Appl. Environ. Microbiol..

[48] Bonfante-Fasolo P., Vian B., Perotto S., Faccio A., Knox J.P. (1990). Cellulose and pectin localization in roots of mycorrhizal *Allium porrum: Labelling* continuity between host cell wall and interfacial material. Planta.

[49] Balestrini R., Hahn M.G., Bonfante P. (1996). Location of cell-wall components in ectomycorrhizae of *Corylus avellana* and *Tuber Magnatum*. Protoplasma.

[50] Volpi C., Janni M., Lionetti V., Bellincampi D., Favaron F., D’Ovidio R. (2011). The Ectopic Expression of a Pectin Methyl Esterase Inhibitor Increases Pectin Methyl Esterification and Limits Fungal Diseases in Wheat. MPMI.

[51] Lionetti V., Giancaspro A., Fabri E., Giove S.L., Reem N., Zabotina O.A., Blanco A., Gadaleta A., Bellincampi D. (2015). Cell wall traits as potential resources to improve resistance of durum wheat against *Fusarium graminearum*. BMC Plant Biol..

[52] Bethke G., Grundman R.E., Sreekanta S., Truman W., Katagiri F., Glazebrook J. (2014). Arabidopsis pectin methylesterases contribute to Immunity Against *Pseudomonas syringae*. Plant Physiol..

[53] Ferrari S., Savatin D.V., Sicilia F., Gramegna G., Cervone F., Lorenzo G.D. (2013). Oligogalacturonides: Plant damage-associated molecular patterns and regulators of growth and development. Front. Plant Sci..

[54] Osorio S., Castillejo C., Quesada M.A., Medina-Escobar N., Brownsey G.J., Suau R., Heredia A., Botella M.A., Valpuesta V. (2008). Partial demethylation of oligogalacturonides by pectin methyl esterase 1 is required for eliciting defence responses in wild strawberry (*Fragaria vesca*). Plant J..

[55] Randoux B., Renard-Merlier D., Mulard G., Rossard S., Duyme F., Sanssene J., Courtois J., Durand R., Reignault P. (2010). Distinct defenses induced in wheat against powdery mildew by acetylated and nonacetylated oligogalacturonides. Phytopathology.

[56] Xu L., Zhu L., Tu L., Liu L., Yuan D., Jin L., Long L., Zhang X. (2011). Lignin metabolism has a central role in the resistance of cotton to the wilt fungus *Verticillium dahliae* as revealed by RNA-Seq-dependent transcriptional analysis and histochemistry. J. Exp. Bot..

[57] Smith A.H., Gill W.M., Pinkard E.A., Mohammed C.L. (2007). Anatomical and histochemical defence responses induced in juvenile leaves of *Eucalyptus globulus* and *Eucalyptus nitens* by *Mycosphaerella* infection. For. Pathol..

[58] Zhang S.H., Yang Q., Ma R.C. (2007). *Erwinia carotovora ssp. carotovora* infection induced “defense lignin” accumulation and lignin biosynthetic gene expression in Chinese cabbage (*Brassica rapa L. ssp. pekinensis*). J. Integr. Plant Biol..

[59] Eynck C., Séguin-Swartz G., Clarke W.E., Parkin I.A. (2012). Monolignol biosynthesis is associated with resistance to *Sclerotinia sclerotiorum* in *Camelina sativa*. Mol. Plant Pathol..

[60] Nawrocka J., Małolepsza U., Szymczak K., Szczech M. (2018). Involvement of metabolic components, volatile compounds, PR proteins, and mechanical strengthening in multilayer protection of cucumber plants against *Rhizoctonia solani* activated by *Trichoderma atroviride* TRS25. Protoplasma.

[61] Underwood W. (2012). The plant cell wall: A dynamic barrier against pathogen invasion. Front. Plant Sci..

[62] Nishimura M.Y., Stein M., Hou B.-H., Vogel J.P., Edwards H., Somerville S.C. (2003). Loss of a Callose Synthase Results in Salicylic Acid–Dependent Disease Resistance. Science.

[63] Durrant W.E., Dong X. (2004). Systemic Acquired Resistance. Annu. Rev. Phytopathol..

[64] Marcus S.E., Verhertbruggen Y., Hervé C., Ordaz-Ortiz J.J., Farkas V., Pedersen H.L., Willats W.G.T., Knox J.P. (2008). Pectic homogalacturonan masks abundant sets of xyloglucan epitopes in plant cell walls. BMC Plant Biol..

[65] Pogorelko G., Lionetti V., Fursova O., Sundaram R.M., Qi M., Whitham S.A., Bogdanove A.J., Bellincampi D., Zabotina O.A. (2013). *Arabidopsis* and *Brachypodium distachyon* transgenic plants expressing *Aspergillus nidulans* acetylesterases have decreased degree of polysaccharide acetylation and increased resistance to pathogens. Plant Physiol..

[66] Moscetti I., Tundo S., Janni M., Sella L., Gazzetti K., Tauzin A., Giardina T., Masci S., Favaron F., D’Ovidio R. (2013). Constitutive Expression of the Xylanase Inhibitor TAXI-III Delays Fusarium Head Blight Symptoms in Durum Wheat Transgenic Plants. Mol. Plant-Microbe Interact..

[67] Berry A.M., Rasmussen U., Bateman K., Huss-Danell K., Lindwall S., Bergman B. (2002). Arabinogalactan proteins are expressed at the symbiotic interface in root nodule of *Alnus* spp.. New Phytol..

[68] van Buuren M.L., Maldonado-Mendoza I.E., Trieu A.T., Blaylock L.A., Harrison M.J. (1999). Novel genes induced during an arbuscular mycorrhizal (AM) symbiosis formed between *Medicago truncatula* and *Glomus versiforme*. Mol. Plant Microbe Interact..

[69] Gaspar Y.M., Nam J., Schultz C.J., Lee L.Y., Gilson P.R., Gelvin S.B., Bacic A. (2004). Characterization of the *Arabidopsis* lysine-rich arabinogalactan-protein *AtAGP17* mutant (rat1) that results in a decreased efficiency of agrobacterium transformation. Plant Physiol..

[70] Buśko M., Chełkowski J., Popiel D., Perkowski J. (2008). Solid substrate bioassay to evaluate impact of *Trichoderma* on trichothecene mycotoxin production by *Fusarium* species. J. Sci. Food Agric..

[71] Popiel D., Kwaśna H., Chełkowski J., Stępień Ł., Laskowska M. (2008). Impact of selected antagonistic fungi on *Fusarium* species—toxigenic cereal pathogens. Acta Mycol..

